# Supplementary material for: Oral Hygiene Practices and Knowledge among Adolescents Aged between 15 and 17 Years Old during Fixed Orthodontic Treatment: Multicentre Study Conducted in France
Source: Int J Environ Res Public Health. 2022 Feb 17;19(4):2316. doi: 10.3390/ijerph19042316 (PMC8872276; doi:10.3390/ijerph19042316)
Supplement: Supplementary file 1 [file ijerph-19-02316-s001.zip › ijerph-1571032-supplementary/ijerph-1571032-supp1.pdf]

Oral hygiene practices and knowledge among adolescents aged between 15 and 17 years old during fixed orthodontic treatment: multicentre study conducted in France

Camille Inquimbert, Celine Clement, Antoine Couatarmanach, Paul Tramini, Denis Bourgeois, Florence Carrouel

Supplementary Table S1. Knowledge of adolescents regarding prevention of oral diseases according to orthodontic treatment. n: number of adolescents.

Variable		No orthodontic treatment (n = 266)	Orthodontic treatment (n = 166)	p-value (*) (chi-square)
Frequency of daily toothbrushing				0.909
	Less than twice	3.6%	4.9%	
	Twice	43.5%	43.3%	
	More than twice	52.9%	51.8%	
Duration of toothbrushing	1 minute or less	0.4%	0.0%	0.231
	2 minutes	6.6%	6.0%	
	3 minutes	21.7%	30.1%	
	> 3 minutes	59.7%	55.4%	
	I don't know	11.1%	7.8%	
Snacking reduces caries				0.330
	No	80.5%	76.5%	
	Yes	9.7%	12.1%	
	I don't know	0%	0%	
Sticky food protects from decay				0.319
	No	92.9%	96.4%	
	Yes	1.8%	1.2%	
	I don't know	5.3%	2.4%	
Fluoride toothpaste prevents calculus				0.578
	No	12.4%	13.3%	
	Yes	37.6%	48.2%	
	I don't know	50.0%	38.6%	
Fluoride toothpaste makes teeth white				0.880
	No	14.6%	18.1%	
	Yes	44.3%	52.4%	
	I don't know	41.2%	29.5%	
Fluoride toothpaste provides fresh breath				0.635
	No	22.6%	24.1%	
	Yes	32.7%	39.8%	
	I don't know	44.7%	36.1%	
Fluoride toothpaste prevents decay				0.001
	No	12.0%	7.2%	
	Yes	46.0%	65.1%	
	I don't know	42.0%	27.7%	

(*) p-values were calculated without considering the answer 'I don't know'

Supplementary Table S2. Oral hygiene, nutrition, and harmful habits among adolescents aged between 15 and 17 years old according to orthodontic treatment. n: number of adolescents.

Variable		No orthodontic treatment (n = 266)	Orthodontic treatment (n = 166)	p-value (*) (chi-square)
Dental floss use				0.635
	Often	0.9%	1.2%	
	Sometimes	7.5%	6.0%	
	Rarely	11.5%	15.7%	
	Never	78.8%	76.5%	
	I don't know	1.3%	0.6%	
Interdental brush use				<0.001
	Often	0.4%	12.1%	
	Sometimes	4.0%	23.0%	
	Rarely	12.0%	13.9%	
	Never	81.9%	48.5%	
	I don't know	1.8%	2.4%	
Frequency of snacking				0.237
	Less than 3 times	2.2%	3.0%	
	3 times	11.1%	11.5%	
	4 times	23.5%	33.1%	
	5 times	23.9%	18.7%	
	More than 5 times	39.4%	33.7%	
Fizzy drinks				0.040
	Often	48.7%	39.8%	
	Sometimes	38.5%	39.2%	
	Rarely	8.9%	18.1%	
	Never	4.0%	3.0%	
	I don't know	0.0%	0.0%	
Sweets				0.167
	Often	33.6%	24.7%	
	Sometimes	38.9%	45.2%	
	Rarely	22.6%	27.1%	
	Never	4.9%	3.0%	
	I don't know	0.0%	0.0%	
Fast foods				0.027
	Often	25.8%	15.1%	
	Sometimes	40.9%	42.2%	
	Rarely	29.3%	34.3%	
	Never	4.0%	8.4%	
	I don't know	0.0%	0.0%	
Cigarettes				0.002
	Often	11.1%	4.2%	
	Sometimes	6.7%	2.4%	
	Rarely	9.8%	4.8%	
	Never	72.4%	88.0%	
	I don't know	0.0%	0.6%	
Drugs				0.422
	Often	0.4%	1.2%	
	Sometimes	3.5%	1.8%	
	Rarely	3.5%	1.8%	
	Never	92.5%	94.6%	
	I don't know	0.0%	0.6%	
Alcohol				0.226
	Often	2.2%	2.4%	
	Sometimes	23.0%	17.5%	
	Rarely	22.6%	17.5%	
	Never	52.2%	62.1%	
	I don't know	0.0%	0.6%	

(*) p-values were calculated without considering the answer 'I don't know'

Supplementary Table S3. Self-perception of oral and general health according to orthodontic treatment. n: number of adolescents.

**Variable**		**No orthodontic treatment (n = 266)**	**Orthodontic treatment (n = 166)**	**p-value (*) (chi-square)**
Difficulty eating				0.068
	Often	4.4%	3.0%	
	Sometimes	11.1%	13.3%	
	Rarely	23.0%	33.7%	
	Never	61.5%	49.4%	
	I don't know	0.0%	0.6%	
Difficulty chewing				0.002
	Often	3.5%	3.0%	
	Sometimes	9.4%	14.5%	
	Rarely	21.0%	34.9%	
	Never	66.1%	47.0%	
	I don't know	0.0%	0.6%	
Toothache				0.152
	Often	6.6%	3.0%	
	Sometimes	17.3%	24.7%	
	Rarely	34.5%	36.8%	
	Never	41.2%	35.5%	
	I don't know	0.4%	0.0%	
Embarrassed about their teeth				0.007
	Often	8.1%	10.3%	
	Sometimes	8.5%	18.8%	
	Rarely	13.8%	15.1%	
	Never	67.4%	55.8%	
	I don't know	2.2%	0.0%	

(*) p-values were calculated without considering the answer 'I don't know'
