# Supplementary material for: Oral Hygiene Practices and Knowledge among Adolescents Aged between 15 and 17 Years Old during Fixed Orthodontic Treatment: Multicentre Study Conducted in France
Source: Int J Environ Res Public Health. 2022 Feb 17;19(4):2316. doi: 10.3390/ijerph19042316 (PMC8872276; doi:10.3390/ijerph19042316)
Supplement: Supplementary file 1 [file ijerph-19-02316-s001.zip › ijerph-1571032-supplementary/ijerph-1571032-supp2.pdf]

Questionnaire on practices and knowledge of oral hygiene

PATIENT CHARACTERISTICS

1. Date of birth I__I__I__I__I
2. Sex ❑ Female ❑ Male
3. How tall are you? **I__I__I__I ft/in** cm
4. How much do you weigh? **I__I__I__I lbs (**Kg)
5. Postal code/ZIP I__I__I__I__I__I
6. Where do you study? ❑Middle School ❑High school ❑Technical High School ❑Other ....❑Don't know
7. What is your father's occupation?..
8. What is your mother's occupation?..
9. Who do you live with? ❑Your two parents ❑Your mother ❑Your father ❑Alternately ❑Other
10. Do you have any siblings? ❑ Yes ❑ No ❑ Don't know

    If "yes", how many? I__I__I

    Which child are you ❑ Eldest(1st) ❑ 2nd ❑ 3rd ❑ 4th ❑ 5th ❑ Other
11. Are you a beneficiary of any of the following?

	Yes	No	Don't know
Universal Health Coverage (CMU in French)	❑	❑	❑
Complementary Health Insurance (ACS in French)	❑	❑	❑
Continuous access to health care (PASS in French)	❑	❑	❑
State medical assistance (AME in French)	❑	❑	❑
Other	❑	❑	❑

CHARACTERISTICS OF THE CONSULTATION

12. When did you last visit a dentist?

    ❑ < 1 year ❑ < 2 years ❑ < 5 years ❑ > or = 5 years ❑Never ❑ Don't know

13. What was the reason for your last dental visit?

    ❑ Checkup, consultation ❑ Cleaning ❑ Emergency

    ❑ Care, followed by regular treatment ❑ Other:...

14. What is the reason for your visit today?

    ❑ Checkup, consultation ❑ Cleaning ❑ Emergency

    ❑ Care, followed by regular treatment ❑ Other:...

15. In the last 12 months, how often have you done the following…?

	Often	Sometimes	Rarely	Never	Don't know
Found it difficult to eat	❑	❑	❑	❑	❑
Had toothache because of sensitive teeth	❑	❑	❑	❑	❑
Had difficulty chewing or biting food	❑	❑	❑	❑	❑
Had toothache	❑	❑	❑	❑	❑
Had bleeding gums	❑	❑	❑	❑	❑
Felt embarrassed because of the appearance of your teeth	❑	❑	❑	❑	❑
Avoided talking because of the appearance of your teeth or denture	❑	❑	❑	❑	❑

ORAL HEALTH, HABITS AND RISK FACTORS

I will now ask you some questions about your dental and food habits…

16. How often do you brush your teeth per day? I__I__I

17. What type of toothbrush do you use?
❑ Manual ❑ Electric ❑ Both ❑ Don't know

18. What are your toothbrush bristles like? ❑ Soft ❑ Medium ❑ Firm ❑ Don't know

19. How often do you replace your toothbrush? ❑ Every 0-3 months❑ 3 - 6 months❑ > 6 months ❑ Don't know

20. What is the name of your toothpaste? …………………………….. ❑ Don't know

21. Do you use…?

	Often	Sometimes	Rarely	Never	Don't know
Dental floss	❑	❑	❑	❑	❑
Interdental brush	❑	❑	❑	❑	❑
Mouthwash	❑	❑	❑	❑	❑

22. Who taught you how to brush your teeth?
❑Parents ❑Dentist ❑School ❑No one ❑Other………………………………… ❑ Don't know

23. How often do you eat/drink per day, even in small amounts? I__I__I

24. At what time(s) do you brush your teeth (multiple answers possible)?
❑Before breakfast ❑After breakfast ❑After lunch
❑After dinner ❑Other (specify):………………. … ❑Don't know

25. About your habits….

	Often	Sometimes	Rarely	Never	Don’t know
Do you ever eat:	❑	❑	❑	❑	❑
Sweet or carbonated drinks?	❑	❑	❑	❑	❑
Chewing gum?	❑	❑	❑	❑	❑
Sweets?	❑	❑	❑	❑	❑
Fast food?	❑	❑	❑	❑	❑
Do you or have you ever smoked cigarettes?	❑	❑	❑	❑	❑
Do you take drugs?	❑	❑	❑	❑	❑
Do you drink alcohol?	❑	❑	❑	❑	❑
Do you exercise?	❑	❑	❑	❑	❑

I will now ask you some questions about products that could improve the health of your teeth. According to you:

26. What is the minimum number of times per day you should brush your teeth? I__I__I

27. Effective teeth brushing should last…

❑< 1 min ❑1 min ❑2 min ❑3 min ❑> 3 min ❑ Don’t know

28. Does snacking between meals reduce the risk of cavities?

❑ Yes ❑ No ❑ Don’t know

29. Does the consumption of sticky foods (chips, sweets, etc.) protect teeth from decay?

❑ Yes ❑ No ❑ Don’t know

30. What is the minimum number of times you should visit a dentist per year? I__I__I

31. Toothpaste should contain fluoride to:

	Yes	No	Don’t know
Prevent the appearance of calculus ?	❑	❑	❑
Make your teeth white?	❑	❑	❑
Have fresh breath?	❑	❑	❑
Reduce the appearance of cavities?	❑	❑	❑

32. Have you ever had any cavities? ❑ Yes ❑ No ❑ Don’t know

33. Are you currently receiving orthodontic treatment? ❑ Yes ❑ No ❑ Don’t know

34. Have you had orthodontic treatment in the past? ❑ Yes ❑ No ❑ Don’t know

35. Have you ever had a consultation with an orthodontist? ❑ Yes ❑ No ❑ Don’t know

QUALITY OF LIFE

36. How do you currently consider the following…?

	Very good	Good	Average	Bad	Very bad	Don’t know
Your general health status	❑	❑	❑	❑	❑	❑
Your dental health status	❑	❑	❑	❑	❑	❑
Your gum health status	❑	❑	❑	❑	❑	❑
